# Bridging Physical and Mental Health: Diabetes Management in Psychiatric Inpatients, a Multidisciplinary Survey

**DOI:** 10.1192/j.eurpsy.2026.11506

**Published:** 2026-07-31

**Authors:** J. Parker, I. Piper

**Affiliations:** NHS Scotland, Edinburgh, United Kingdom

## Abstract

**Introduction:**

Individuals with severe mental illness (SMI) have a life expectancy 10-to-20-years shorter than the general population. Increased morbidity and mortality is frequently due to preventable physical health conditions. In diabetes, those with an SMI have significantly worse health outcomes. These outcomes are particularly apparent in inpatient groups, where it is estimated that 10% have Type 2 Diabetes (T2DM) and 1% have Type 1 Diabetes (T1DM). Managing diabetes in psychiatric inpatients is challenging, and integration of diabetic care into psychiatric care remains inconsistent. Inpatient admission is a missed opportunity for improving the diabetic care of those with SMI.

**Objectives:**

The goal of this survey was to understand current practices, confidence levels, and perceived barriers when managing diabetes across the multidisciplinary team at the Royal Edinburgh Hospital (REH), a large psychiatric hospital in Scotland. These insights can be used to improve staff education, guidance and patient care.

**Methods:**

A 13-item questionnaire was used to evaluate healthcare professionals’ understanding, and confidence in managing diabetes in an inpatient psychiatric setting. Participants were also asked to evaluate their perception of the quality of care provided to inpatients. Participants included all staff authorised to prescribe or dispense insulin. The questionnaire was sent via an anonymous electronic survey to all hospital subspecialities.

**Results:**

69 responses were collected from six subspecialties across the REH: 42 nurses, 24 doctors, and 3 allied healthcare workers. Participants agreed they were confident in monitoring blood glucose (81% - 56/69), treating low blood sugar (88% - 61/69), treating high blood sugar (80% - 55/69), understanding the difference between T1DM and T2DM (83% - 57/69), and understanding the difference between long and short acting insulin (68% - 47/69). However, only 33% (23/69) agreed that the quality of diabetic care that patients received on the ward was of an acceptable standard. Respondents noted limited access to specialist services and resources. Only 14% (10/69) had received any training in the management of diabetes in the past 12 months. Lack of training was highlighted in comments. For example, one respondent wrote *“for a type 1 diabetic, I feel there is little to no training”.*

**Conclusions:**

Staff generally agreed that they were confident in managing diabetes. However, concerns were raised about lack of staff training and expert support. There was a widespread perception that inpatient diabetic care was of an unacceptable standard. Our study recommends improving staff education, developing psychiatric setting–specific diabetes protocols, and establishing links with diabetes specialist teams.

**Disclosure of Interest:**

None Declared

